# Improved potassium reliability in whole blood through hemolysis detection on the novel GEM Premier 7000 blood gas analyzer

**DOI:** 10.5937/jomb0-58604

**Published:** 2025-07-04

**Authors:** Laura Pighi, Gian Luca Salvagno, Filippo Marcazzan, Mariateresa Rizza, Giuseppe Lippi

**Affiliations:** 1 Section of Clinical Biochemistry, University of Verona, Verona, Italy

**Keywords:** hemolysis, blood gas analysis, point of care testing, preanalytical variability, hemoliza, analiza gasova krvi, testiranje na mestu nege, preanalitička varijabilnost

## Abstract

**Background:**

Hemolysis is the most frequent preanalytical error in clinical laboratories, but its detection in point-of-care (POC) settings remains challenging due to the lack of sample separation. This study was planned to validate the hemolysis index (HI) threshold of GEM Premier 7000 blood gas analyzer for detecting hemolysis levels that may generate clinically significant interference in potassium measurement.

**Methods:**

Heparinized whole blood samples were collected from healthy volunteers and divided into six aliquots; one was used as non-hemolyzed control, while hemolysis was mechanically induced in the remaining five aliquots by repeated aspirations through a fine-gauge needle. HI and potassium were measured on GEM Premier 7000.

**Results:**

The final study population consisted of 18 volunteers. Both HI and potassium levels increased progressively and significantly with the number of fine-needle aspirations (p<0.001 for both). A strong positive correlation was observed between HI values and percentage increases in potassium concentration in hemolyzed aliquots (r=0.985, p<0.001). Receiver operating characteristic (ROC) curve analysis confirmed excellent diagnostic accuracy of HI in detecting potassium increases above the minimum total 7.4% allowable error threshold, with an area under the curve (AUC) of 1.00 and optimal cutoff of 102 mg/dL (0.94 sensitivity and 1.00 specificity). At the manufacturer-recommended 116 mg/dL threshold, the AUC was 0.95, with 0.89 sensitivity and 1.00 specificity.

**Conclusions:**

These results confirm that the novel GEM Premier 7000 blood gas analyzer provides accurate detection of hemolysis thresholds in whole blood that may impair potassium test reliability.

## Abstract (sr)

**Uvod:**

Hemoliza je najčešća preanalitička greška u kliničkim laboratorijama, ali njeno otkrivanje na mestu lečenja (POC) ostaje izazovno zbog nedostatka odvajanja uzoraka. Ova studija je planirana da potvrdi prag indeksa hemolize (HI) GEM Premier 7000 analizatora gasova u krvi za otkrivanje nivoa hemolize koji mogu da generišu klinički značajne smetnje u merenju kalijuma.

**Metode:**

Uzorci heparinizovane pune krvi su sakupljeni od zdravih dobrovoljaca i podeljeni u šest alikvota; jedan je koriišćen kao nehemolizovana kontrola, dok je hemoliza mehanički indukovana u preostalih pet alikvota ponovljenim aspiracijama kroz iglu finog kalibra. HI i kalijum su mereni na GEM Premier 7000.

**Rezultati:**

Konačnu populaciju studije činilo je 18 dobrovoljaca. I nivoi HI i kalijuma su se progresivno i značajno povećavali sa brojem aspiracija tankom iglom (p<0,001 za oba). Uočena je jaka pozitivna korelacija između vrednosti HI i procentualnog povećanja koncentracije kalijuma u hemolizovanim alikvotima (r=0,985, p<0,001). Analiza krive radne karakteristike prijemnika (ROC) potvrdila je odličnu dijagnostičku tačnost HI u detekciji povećanja kalijuma iznad ukupnog dozvoljenog praga greške od 7,4%, sa površinom ispod krive (AUC) od 1,00 i optimalnom graničnom vrednostom od 102 mg/dL (0,94 osetljivost i 1,00). Na pragu od 116 mg/dL koji je preporučio proizvo|ač, AUC je bio 0,95, sa osetljivošću 0,89 i specifičnošću 1,00.

**Zaključak:**

Ovi rezultati potvr|uju da novi GEM Premier 7000 analizator gasova krvi omogućava precizno otkrivanje pragova hemolize u punoj krvi koji mogu umanjiti pouzdanost testa kalijuma.

## Introduction

Spurious hemolysis, which is conventionally defined as mechanical damage to blood cells (especially erythrocytes) during sample collection and handling, is the leading cause of sample rejection across all clinical laboratories worldwide [1]. Detection of hemolysis is relatively straightforward in serum or plasma, as the presence of cell-free hemoglobin confers a characteristic and visible discoloration to the sample matrix, and can now also be automatically detected and quantified by many modern clinical chemistry and coagulation analyzers [2]. However, identifying hemolysis in whole blood remains analytically challenging, posing a significant risk for preanalytical errors, especially due to its marked impact on potassium levels [3] [4]. Potassium can be abundantly released from lysed erythrocytes and other blood cells, leading to falsely elevated results that may compromise clinical interpretation and patient care [5]. The development and commercialization of blood gas analyzers equipped with dedicated hemolysis detection modules has substantially improved this scenario [6]. Therefore, this study specifically aims to validate the hemolysis index (HI) threshold of the novel GEM Premier 7000 blood gas analyzer (Werfen, Bedford, USA), which incorporates an automated flagging system to assess the reliability of potassium measurements in whole blood samples with clinically significant hemolysis.

## Materials and methods

The hemolysis detection module integrated into the GEM Premier 7000 has been previously described in detail elsewhere [6]. Briefly, this system combines acoustofluidic separation with photometric analysis to detect hemolysis directly in whole blood samples. Acoustic energy is applied to separate plasma from cellular components, enabling optical absorbance measurements at 570 nm and 610 nm in the plasma phase. The resulting HI is expressed in arbitrary units (AU) and classified in six categories of cell-free hemoglobin concentrations (e.g., 0–50, 51–115, 116-200, 201–300, 301–400, and 401 mg/dL). Potassium results are flagged when the HI exceeds a predefined interference threshold of 116 mg/dL. For this evaluation, Werfen provided access to the corresponding quantitative (continuous) HI values, in addition to the standard categorical classifications.

Heparinized whole blood samples (6.0 mL lithium-heparin tubes; Vacutest Kima, Padova, Italy) were collected from 26 healthy volunteers recruited from the laboratory staff. Each sample was divided into six identical aliquots. The first aliquot remained unaltered and served as the baseline control, while hemolysis was mechanically induced in the remaining five aliquots by performing one to five sequential aspirations through a 25-gauge needle attached to an insulin syringe, according to a previously validated protocol [7]. All aliquots were immediately analyzed using the GEM Premier 7000 for measurement of both HI and potassium. The degree of hemolysis-induced potassium increase was expressed as a percentage relative to the baseline value. The accuracy of the HI to detect potassium increases exceeding the minimum total allowable error (TAE) of 7.4%, as defined by the European Federation of Clinical Chemistry and Laboratory Medicine (EFLM) Biological Variation Data base [8], was assessed by receiver operating characteristic (ROC) curve analysis. The minimum TAE derived from biological variability was chosen to interpret variations in whole blood potassium due to preanalytical factors (i.e., sample hemolysis) because it reflects the threshold beyond which clinical interpretation may be affected. In contrast, optimal performance specifications may detect statistically significant but clinically insignificant changes, making them less suitable for assessing the clinical impact of these variations. Additional statistical analyses included the Friedman test and Spearman’s rank correlation to evaluate the relationship between percent potassium increase and HI across the hemolyzed aliquots. The study was conducted in agreement with the Declaration of Helsinki, under the conditions of relevant local legislation and was cleared by the local Ethical Committee (Verona and Rovigo provinces; protocol number: 971CESC, date of approval: 25 July, 2016).

## Results

**Figure 1:**
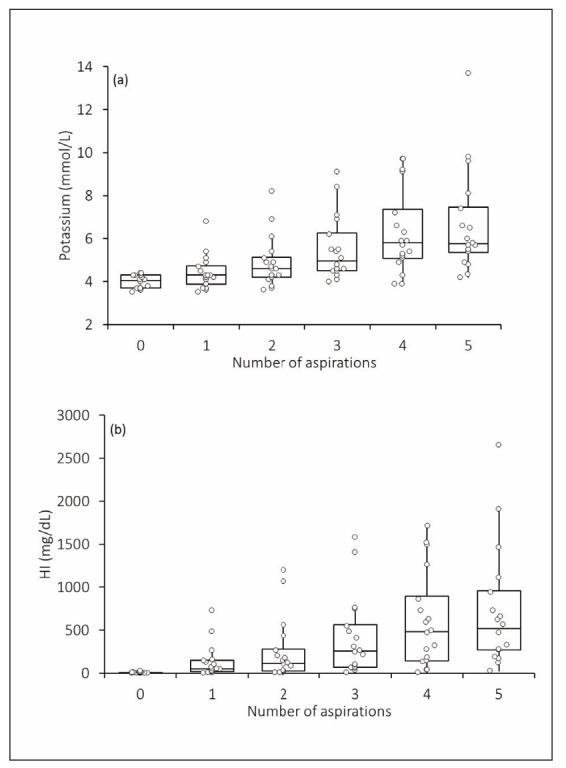
Increase in (a) potassium and (b) hemolysis index (HI) measured by the GEM Premier 7000 with increasing number of fine needle aspirations of heparinized samples. HI, hemolysis index

**Figure 2:**
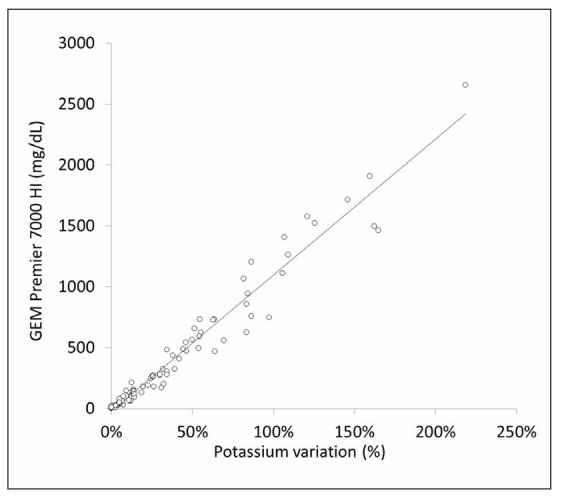
Correlation between the hemolysis index (HI) and the percentage increase in potassium concentration in hemolyzed aliquots measured by the GEM Premier 7000. HI, hemolysis index

The final study population consisted of 18 healthy volunteers, following the exclusion of 8 individuals due to at least one HI value in their hemolyzed aliquots exceeding the analytical measuring range of the GEM Premier 7000 blood gas analyzer. A progressive and statistically significant increase in both potassium concentration (Friedman test statistic: 57.30; p<0.001) and HI (Friedman test statistic: 56.90; p<0.001) was observed with an increasing number of fine needle aspirations (Figure 1). HI values demonstrated a strong positive correlation with the percentage increase in potassium concentration in hemolyzed samples (r=0.985; 95% CI, 0.977-0.990; p<0.001) (Figure 2).

**Figure 3:**
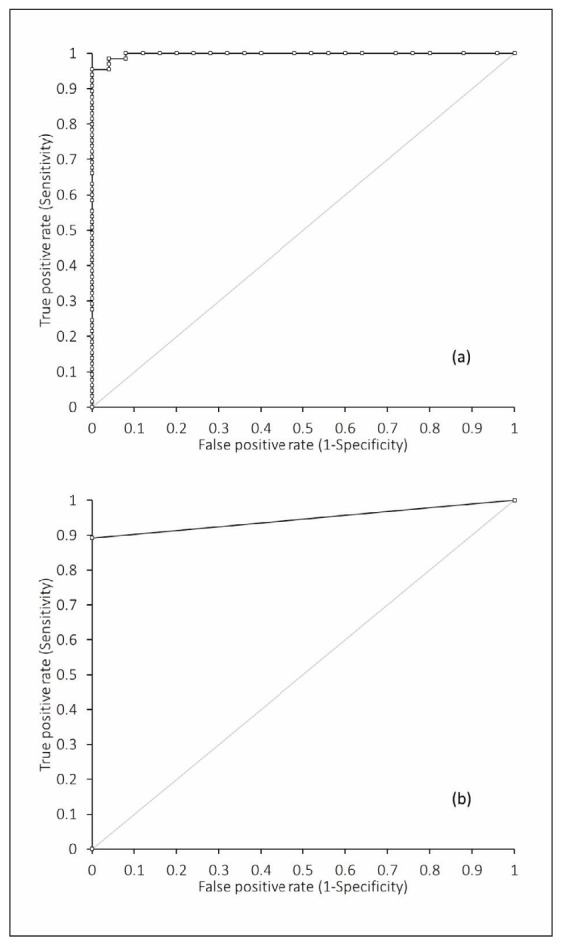
Receiver operating characteristic (ROC) curve analysis for evaluating the performance of the hemolysis index (HI) on GEM Premier 7000 in detecting potassium increases in hemolyzed aliquots exceeding the total allowable error of 7.4%, using (a) continuous HI values and (b) the manufacturer recommended threshold of 116 mg/dL.

The ROC curve analysis showed that the HI, when assessed as continuous variable, exhibited excellent diagnostic accuracy for detecting hemolysis-induced potassium increases exceeding the minimum TAE threshold of 7.4%, with an area under the curve (AUC) of 1.00 (95% CI, 0.99–1.00; p<0.001) (Figure 3a). We found that the optimal HI cutoff for identifying clinically significant hemolysis was 102 mg/dL, corresponding to a sensitivity of 0.94 (95% CI, 0.85–0.98) and a specificity of 1.00 (95% CI, 0.86–1.00). At the manufacturer-recommended threshold of 116 mg/dL, the AUC was 0.95 (95% CI, 0.91–0.98; p<0.001), with sensitivity and specificity of 0.89 (95% CI, 0.79–0.96) and 1.00 (95% CI, 0.86–1.00), respectively (Figure 3b).

## Discussion

Hemolysis in samples for blood gas analysis is a relatively common preanalytical issue, with a reported prevalence ranging from 1% to 13% across different studies [9] [10] [11] [12]. This phenomenon presents a significant challenge to both laboratory professionals and clinicians, as damage of erythrocytes and other blood cells leads to the release of intracellular contents, especially potassium, into the surrounding blood, thus impairing the accuracy of several measured parameters, jeopardizing the clinical reliability of test results and potentially leading to inappropriate patient care.

The detection of hemolysis in whole blood samples, especially in point-of-care (POC) settings such as emergency departments or intensive care units (ICUs), remains particularly problematic. Traditional methods, such as post-analytical centrifugation of the specimen to visually or spectrophotometrically assess plasma discoloration, are impractical in acute care settings due to their time-consuming and labor-intensive nature. Other proposed solutions include the use of external POC devices capable of detecting hemolysis without centrifugation [11], but these introduce additional workflow complexity and cost, often limiting their feasibility in high-throughput, resource-constrained environments.

In this context, the commercialization of blood gas analyzers equipped with built-in and rapid hemolysis detection capabilities, such as the GEM Premier 7000, represents a potentially transformative advancement. This analyzer incorporates an automated HI measurement, delivers results in approximately 45 sec without additional preparation steps, thus potentially offering a practical, time-efficient, and cost-effective solution, provided that its analytical performance is confirmed and validated for clinical use.

In our evaluation, the GEM Premier 7000 demonstrated optimal accuracy in detecting clinically relevant hemolysis in whole blood samples. The HI generated by the analyzer displayed a strong positive correlation with the hemolysis-induced increase in potassium concentration. Both the optimal threshold identified through ROC analysis (102 mg/dL) and the manufacturer-recommended cutoff (116 mg/dL) exhibited high sensitivity and almost perfect specificity for identifying a hemolysis degree likely to be associated with unreliable potassium measurements.

In conclusion, the results of our study support the clinical utility of the GEM Premier 7000 automated hemolysis detection feature. By enabling rapid and reliable identification of hemolyzed specimens at the POC, this technology helps prevent the reporting and clinical use of spuriously elevated potassium values, ultimately enhancing patient safety and supporting more accurate and timely decision-making in critical care settings.

## Dodatak

### Conflict of interest statement

All the authors declare that they have no conflict of interest in this work.
